# Modeling *Agrobacterium*-Mediated Gene Transformation of Tobacco (*Nicotiana tabacum*)—A Model Plant for Gene Transformation Studies

**DOI:** 10.3389/fpls.2021.695110

**Published:** 2021-07-23

**Authors:** Gniewko Niedbała, Mohsen Niazian, Paolo Sabbatini

**Affiliations:** ^1^Department of Biosystems Engineering, Faculty of Environmental and Mechanical Engineering, Poznań University of Life Sciences, Poznań, Poland; ^2^Field and Horticultural Crops Research Department, Kurdistan Agricultural and Natural Resources Research and Education Center, Agricultural Research, Education and Extension Organization, Sanandaj, Iran; ^3^Department of Horticulture, Plant and Soil Sciences Building, Michigan State University, East Lansing, MI, United States

**Keywords:** acetosyringone, artificial neural networks, genetic engineering, inoculation duration, optical density

## Abstract

The multilayer perceptron (MLP) topology of an artificial neural network (ANN) was applied to create two predictor models in *Agrobacterium*-mediated gene transformation of tobacco. *Agrobacterium*-mediated transformation parameters, including *Agrobacterium* strain, *Agrobacterium* cell density, acetosyringone concentration, and inoculation duration, were assigned as inputs for ANN–MLP, and their effects on the percentage of putative and PCR-verified transgenic plants were investigated. The best ANN models for predicting the percentage of putative and PCR-verified transgenic plants were selected based on basic network quality statistics. *Ex-post* error calculations of the relative approximation error (RAE), the mean absolute error (MAE), the root mean square error (RMS), and the mean absolute percentage error (MAPE) demonstrated the prediction quality of the developed models when compared to stepwise multiple regression. Moreover, significant correlations between the ANN-predicted and the actual values of the percentage of putative transgenes (*R*^2^ = 0.956) and the percentage of PCR-verified transgenic plants (*R*^2^ = 0.671) indicate the superiority of the established ANN models over the classical stepwise multiple regression in predicting the percentage of putative (*R*^2^ = 0.313) and PCR-verified (*R*^2^= 0.213) transgenic plants. The best combination of the multiple inputs analyzed in this investigation, to achieve maximum actual and predicted transgenic plants, was at *OD*_600_ = 0.8 for the LB4404 strain of *Agrobacterium* × 300 μmol/L acetosyringone × 20 min immersion time. According to the sensitivity analysis of ANN models, the *Agrobacterium* strain was the most important influential parameter in *Agrobacterium*-mediated transformation of tobacco. The prediction efficiency of the developed model was confirmed by the data series of *Agrobacterium*-mediated transformation of an important medicinal plant with low transformation efficiency. The results of this study are pivotal to model and predict the transformation of other important *Agrobacterium*-recalcitrant plant genotypes and to increase the transformation efficiency by identifying critical parameters. This approach can substantially reduce the time and cost required to optimize multi-factorial *Agrobacterium*-mediated transformation strategies.

## Introduction

A rapid improvement in the important economic traits of plants is needed due to climate change and the steady increase in global population. Nowadays, *in vitro*-based biotechnological methods are applied for breeding with the aim of improving plant genotypes through rapid multiplication, micropropagation of disease-free plants, production of plant-derived metabolites, and gene transformation (Hesami et al., 2020b). Genetic transformation (genetic engineering) is one of the key biotechnological tools to improve plant performance.

The genetic transformation of plantscan be achieved by direct and indirect methods (Niazian et al., 2017). The most effective and well-known laboratory method for indirect gene transfer in plants is through *Agrobacterium* infection (Meyers et al., 2010). The *Agrobacterium* method is a simple, efficient, and practical protocol for the transfer of foreign DNA and is the first prerequisite to produce genetically modified plants (Abbasi et al., 2020). However, this is challenging because of the low efficiency in most of the important plants, as many factors may affect this process. The *Agrobacterium* strain, *Agrobacterium* cell density, immersion time, type and concentration of antibiotics to kill *Agrobacterium*, type and concentration of the selected antibiotics, concentration of acetosyringone, duration of co-cultivation, pH and temperature of co-cultivation, and wounding treatments are the key factors that can affect *Agrobacterium*-mediated gene transformation and should be taken into account in all gene delivery studies (Liu et al., 2020). The first group of factors that affects *Agrobacterium*-mediated gene delivery are *Agrobacterium* strain, *Agrobacterium* cell density, and antibiotic eliminating *Agrobacterium*, while the second group of influential factors is explant type and age along with immersion time and wounding treatment. The third group of significant factors is the concentrations of other additives, such as the selected antibiotics for the plant along with the chemical stimulants. Finally, the fourth group of factors that is involved in *Agrobacterium*-mediated gene transformation which can affect its efficiency are the co-cultivation parameters, such as duration, pH, and temperature. Plasmids for optimizing expression in plants (sub-optimal promoter, enhancer, poor codon usage, 5'UTR sequence, trigger silencing, integration of the gene into a silent region of chromatin) are another important influential factor for the genetic transformation of plants. Numerous studies are underway in order to increase the efficiency of *Agrobacterium*–mediated gene transformation in different plant species and genotypes by optimizing the aforementioned parameters. In ajowan (*Trachyspermum ammi* L.), a medicinal plant, different levels of gene transformation parameters, including the *Agrobacterium* optical density (OD), *Agrobacterium* strain, *Agrobacterium* killing antibiotic, acetosyringone concentration, and inoculation duration were assessed during the introduction of the *BADH* gene, and greatest gene transformation efficiency was obtained using the LB4404 strain of *Agrobacterium* at *OD*_600_= 0.6–0.8 × 160 mg/L timentin × 250 μmol/L acetosyringone × 30 min inoculation duration (Niazian et al., 2019). The different levels of cell density of *A. tumefaciens* (*OD*_600_ = 0.2, 0.3, 0.5, 0.8, 1.0, 1.2, 1.4) and the concentrations of acetosyringone (0–100 μM) were investigated in *Agrobacterium*-mediated gene transformation of *Veratrum dahuricum*, a medicinal plant. An optical cell density of 0.8 (*OD*_600_ = 0.8) at 600 nm along with 20 μM of acetosyringone were reported as the optimum levels for these parameters (Ma et al., 2020). Different suspension solutions (*OD*_600_ = 0.2, 0.4, 0.6, 0.8, and 1.0), along with the immersion durations (10, 20, 30, and 40 min) and acetosyringone concentrations (50, 100, 150, and 200 μM), were investigated in *Agrobacterium*-mediated gene transformation of *Pinus tabuliformis* and at 600 nm for *Agrobacterium*, an optical density of 0.8 × 150 μM acetosyringone × 30 min immersion time were reported as the optimal gene transformation factors (Liu et al., 2020). The effects of various optical densities of the *Agrobacterium* suspension (*OD*_600_ = 0.3, 0.35), duration of incubation (5, 10, 15, 20, and 25 min), and co-cultivation time (24, 48, 72, 96, and 120 h) were investigated in the *Agrobacterium*-mediated transformation efficiency of pigeon pea [*Cajanus cajan* (L.) Millsp] (Karmakar et al., 2019). The authors reported a transformation efficiency of 83% using *Agrobacterium* cells at an optical density (OD_600_) of 0.25, with an immersion time of 15 min, co-culturing with explants for 72 h which served as the optimized parameters of transformation (Karmakar et al., 2019). The different levels of acetosyringone concentration (0, 50, 100, 150, and 200 mM) and *A. tumefaciens* cell density (*OD*_600_ = 0.2, 0.4, 0.6, 0.8, and 1.0) were assessed to improve the *Agrobacterium*-mediated transformation efficiency of cotton (*Gossypium hirsutum* L.‘KC3') and an *A. tumefaciens* cell suspension of OD_600_ nm = 0.6 containing 100 mM acetosyringone led to the maximum transformation efficiency of 20.25% (Gurusaravanan et al., 2020).

The incorporation of different chemicals and additives into the medium, which trigger the transformation activity of *Agrobacterium* or increase the regeneration efficiency, is the next solution to increase the efficiency of *Agrobacterium*-mediated transformation. In lily (*Lilium cv*‘Manissa') as a cut flower, a higher transformation efficiency (11.1%) was achieved when chloroxynil was used instead of acetosyringone (6.6%) as the phenolic compound (Abbasi et al., 2020). In soybean [*Glycine max* (L.) Merrill], the transformation efficiency was improved (34.6% vs. 23%) when sodium nitroprusside (as the leading source of the nitric oxide donor) was applied in both shoot inducing and rooting media (Karthik et al., 2020). Spermidine incorporation in the culture medium (15 mg/L), as a polyamine, led to the increased *Agrobacterium*-mediated transformation efficiency (17.3%) in watermelon (*Citrullus lanatus* Thunb.) *cv*. Arka Manik (Vasudevan et al., 2020).

The interaction between the plant genotype and the aforementioned factors, challenges the implementation of the transformation strategies, leading to genotype-dependency in gene transformation studies; meaning the different responses of different plant genotypes to a specific protocol. As an *in vitro* procedure, *Agrobacterium*-mediated transformation is a multi-factorial biological system that is highly variable and complex in nature, making it a non-deterministic and a non-linear process. Highly non-linear and complex relationships of biological events are difficult to predict by regression-based models. Analysis and interpretation of non-linear biological systems by non-linear and non-parametric methods is much more efficient. One of the powerful non-linear and non-parametric computational methods to overcome the problem of regression models is the artificial neural network (ANN) (Emamgholizadeh et al., 2015; Pentoś, 2016). A good illustration of this approach is the work conducted by Wawrzyniak (Wawrzyniak, 2020), which demonstrates the superiority of neural networks over a traditional regression-based model in describing the dynamics of fungal growth in the mass of stored rapeseeds. The author reported that the use of neural networks not only allows models that better describe the phenomenon but are also, due to the lack of preliminary assumptions, able to use the wide spectrum of experimental data that could not be used in non-linear regression. Artificial neural networks have been successfully used to analyze non-linear relationships prevalent in a variety *in vitro* studies in plants (Dutta Gupta and Pattanayak, 2017; Arab et al., 2018). Tobacco is a model plant for gene transformation studies (Mushtaq et al., 2020). Modeling and optimizing the gene transformation protocol in this plant can encourage researchers to establish efficient protocols in other desired recalcitrant plant species. The aim of this study was to create two predictive models in *Agrobacterium*-mediated gene transfer of tobacco under the influence of different gene transformation parameters.

## Materials and Methods

### The Agrobacterium-Mediated Gene Transformation Procedure and Calculation of the Percentage of Putative and PCR-Verified Transgenic plants

**Figure 1:**
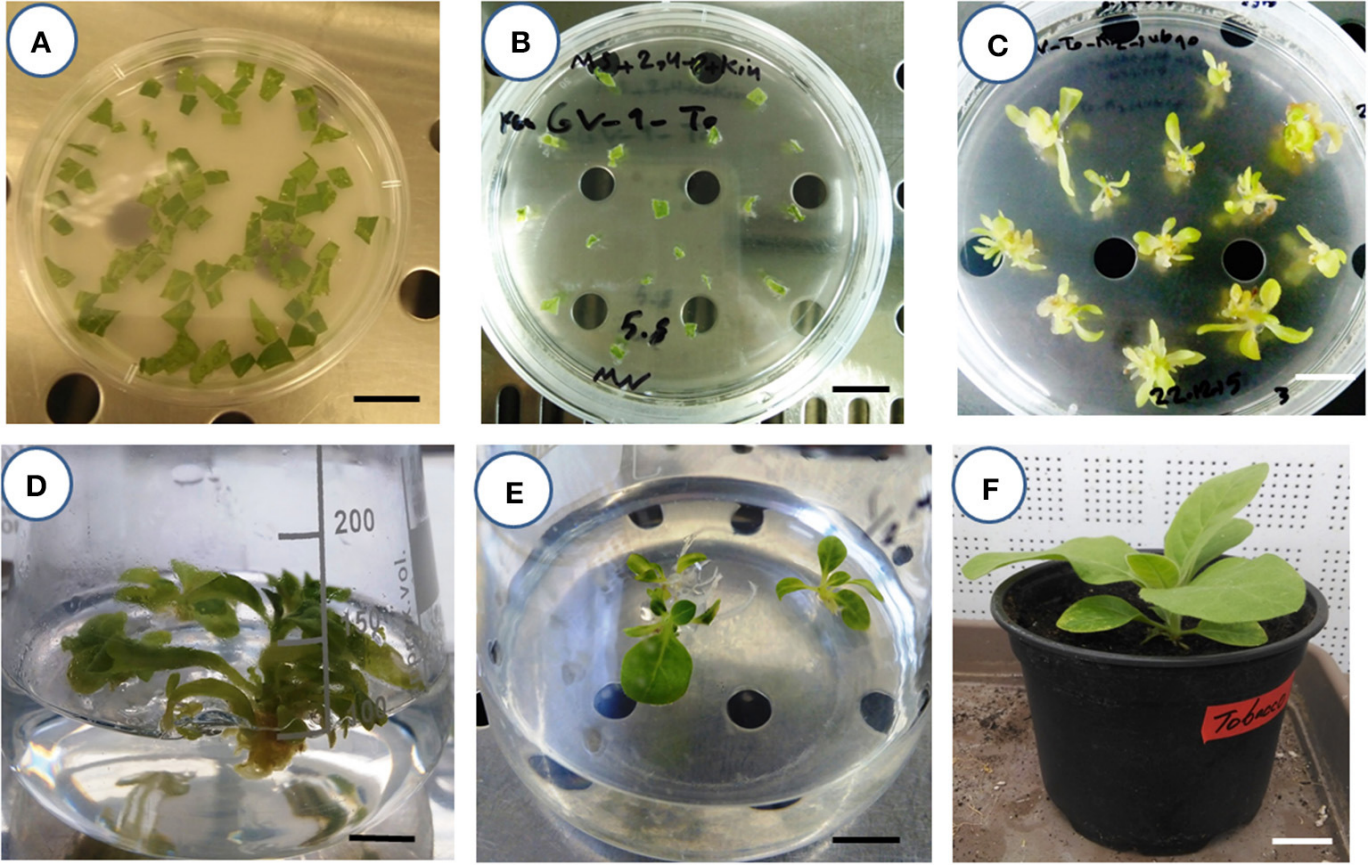
Steps for creating putative transgenic tobacco plants. **(A)** The inoculation of leaf square explants in *Agrobacterium* suspension (bar = 2 cm). **(B)** The establishment of *Agrobacterium*-inoculated explants in an agar-solidified selective regeneration medium (bar = 2 cm). **(C)** The emergence of direct regenerated shoots in the selective regeneration medium (bar = 2 cm). **(D)** The rooting of putative transgenic shoots in the PGR-free MS medium containing kanamycin and cefotaxime antibiotics (bar = 2 cm). **(E)** The elongation of the rooted putative transgenic seedling of tobacco in the PGR-free MS medium containing kanamycin and cefotaxime antibiotics (bar = 2 cm). **(F)** The acclimatization of the putative transgenic tobacco plant under greenhouse conditions (bar = 4 cm).

A routine *Agrobacterium*-mediated gene delivery of tobacco was conducted using the pCAMBIA2301 binary vector (11634 bp). This vector carries the kanamycin resistance gene of *neomycin phosphotransferase* (*nptII*), which acts as a selectable marker for plant selection, and the *gusA* reporter gene. In the left border (LB), the *nptII* gene is driven by the cauliflower mosaic virus 35S (CaMV35S2) promoter and CaMV35S terminator, whereas the intron—*gusA* in the right border (RB) is driven by the CaMV35S promoter and NOS-terminator. The effects of the factors in *Agrobacterium*-mediated *gene* transformation, including *Agrobacterium* strains (AGL1, LB4404, and GV3101), *Agrobacterium* cell density (*OD*_600_ = 0.6, 0.7, and 0.8), acetosyringone concentration (200, 300, and 400 μmol/L), and inoculation duration (immersion time) (1, 10, and 20 min) were assessed. Pre-incubated (2 days) leaf disk explants (1 × 1 cm^2^) of 4-week-old *in vitro*-obtained plantlets of tobacco were inoculated into the *Agrobacterium* suspension, containing the binary vector, were blot dried on sterile filter paper and co-cultivated in a phytotron at 25 ± 1°C with 60 to 70% relative humidity for 48 h (in dark). The Murashige and Skoog medium (MS) (Murashige and Skoog, 1962) supplemented with 0.1 mg/L indole-3-acetic acid (IAA) + 1 mg/L 6-benzylaminopurine (6-BA) and 100 μM acetosyringone was used for co-cultivation (Pathi et al., 2013; Leng et al., 2020). Explants were then transferred to the selective regeneration medium. An agar-solidified MS medium, supplemented with 1 mg/L 2,4-Dichlorophenoxyacetic acid (2,4-D) + 0.5 mg/L kinetin (Kin) + 200 mg/L kanamycin + 160 mg/L ticarcillin disodium/clavulanate potassium was used for direct shoot regeneration. All cultures were maintained in a phytotron at 25 ± 1°C with 60 to 70% relative humidity under a 16/8 (light/dark) photoperiod. Regular subcultures of explants were performed in the same medium. Then, the emerging shoots (15th day of the experiment) were transferred to a PGR-free MS medium, supplemented with 100 mg/L kanamycin + 300 mg/L cefotaxime, for rooting, maturation, and elongation. After 2 weeks, the roots of the plantlets were washed with distilled water to remove any traces of agar. Then, plantlets were transferred to plastic pots (200-ml) containing autoclaved perlite:peat moss (1:1) and were kept in a phytotron, with aforementioned conditions, for 7 days. The surviving plantlets were assumed as putative transgenic plants and were used as the first dependent variable (output) of the experiment. Figures 1A–F reports all the laboratory steps, from inoculation of the explants to the acclimatization of the putative transformed tobacco plants. Basal MS medium, plant agar, all PGRs, and antibiotics were supplied by Duchefa (Haarlem, The Netherlands). The genomic DNA of putative transformed (kanamycin-resistant) plants was extracted using the method described by Sika et al. (2015). The specific primers (forward: 5′-CCACCATGATATTCGGCAAC-3′ and reverse: 5′-GTGGAGAGGCTATTCGGCTA-3′) were used for the amplification of the *NPTII* gene (0.54 kb fragment) with polymerase chain reaction (PCR). The amplification was performed using a thermal cycler (MyCycler, BIO-RAD, USA) under the conditions described by Saini and Sonia (2003). The PCR products were separated by electrophoresis on a 1% agarose gel and visualized by ethidium bromide. The percentage of PCR- positive plants were recorded and used as output for the second predictor model.

### Construction of Artificial Neural Networks and Statistical Analysis

**Figure 2:**
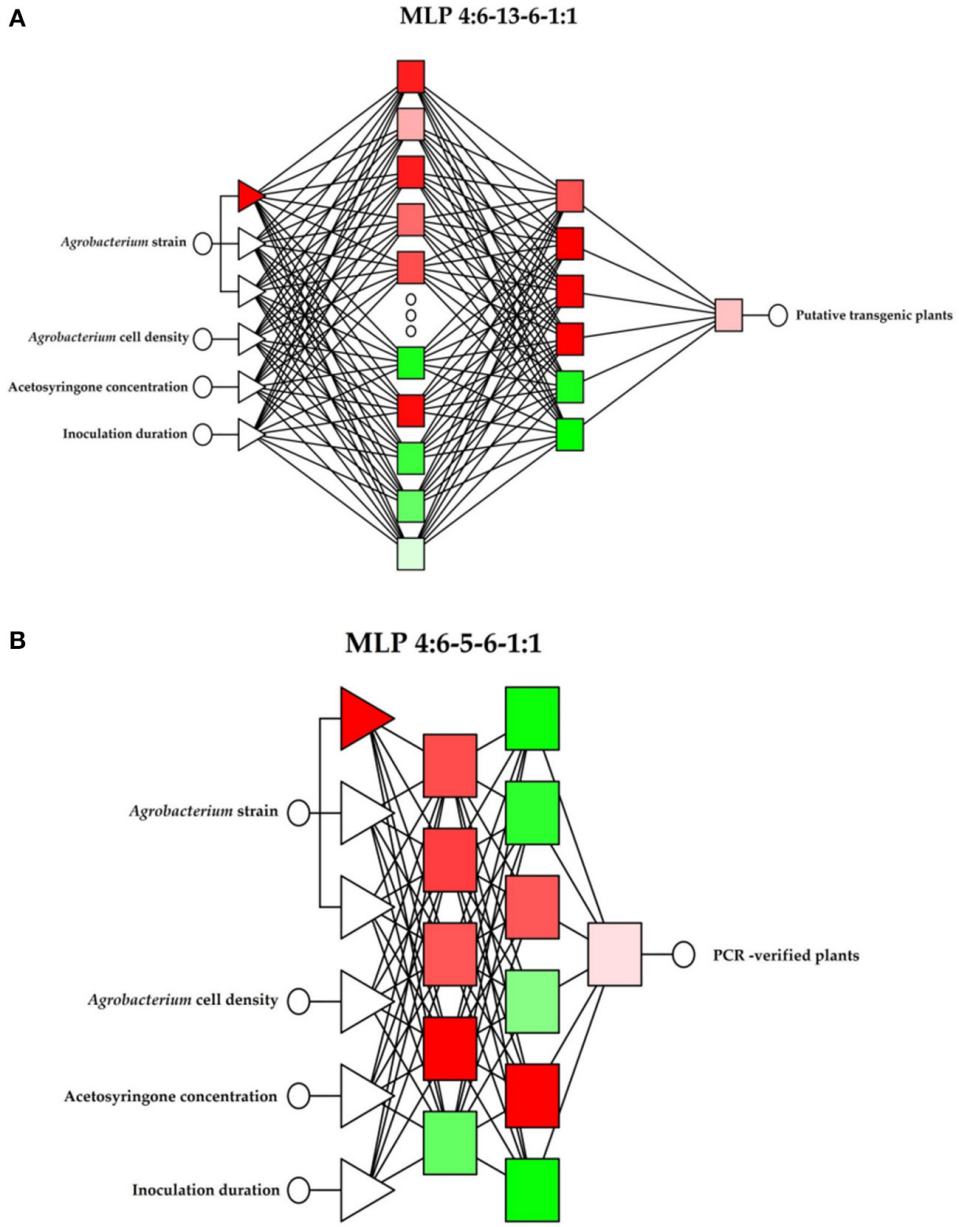
The topology of applied ANN models to predict the percentage of putative and PCR-verified transgenic tobacco. **(A)** The topology of the ANN with four input factors to predict the percentage of putative transgenic tobacco plants. **(B)** The topology of the ANN with four input factors to predict the percentage of PCR-verified plants.

A factorial experiment based on a completely randomized design with three replications (Petri dishes) was carried out to study the combined effect of independent variables (number of independent observations = 3 × 3 × 3 × 3 × replications = 243). Five explants were cultured in each Petri dish and the percentage of putative transgenes and PCR-verified plants were considered as dependent variables (y_1_, y_2_). The ANOVA for the number of putative and PCR-verified transgenic plants was performed using SAS® (SAS Institute Inc., Cary, NC, USA) software. Two MLP topologies of the ANN (Kujawa et al., 2014), each with two hidden layers containing neurons, was trained and tested to assess the effect of independent variables (*Agrobacterium* strains, *Agrobacterium* cell density, acetosyringone concentration, and inoculation duration) on dependent variables (percentage of putative and PCR-verified plants). The first of the independent variables was qualitative and involved the three adopted values of the feature considered. The other independent values were quantitative. The extended ANN topologies, including input, output, and number of hidden layers, for percentage of putative transgenic plants and PCR-verified plants are presented in Figures 2A,B, respectively. In both models, the independent variables were directly fed into the input layer and their interaction effects on the output were evaluated. An Automatic Network Designer (AND) from Statistica v7.1 (StatSoft Inc., Tulsa, OK, USA) was applied to build the ANN models. The following assumptions were made when using the AND: MLP structure of the neural network; 1 or 2 hidden layers; 1–20 neurons in each hidden layer; activation function—linear or logistic. The optimization process was conducted using trial and error to find the best topology of the AND: MLP structure. In both models, the experimental data (243 data) were divided into 70%:15%:15% parts for training, validation, and testing, respectively. Basic network quality statistics, including SD, mean error, deviation error, mean absolute error, quotient of deviation, and correlation coefficient, were calculated and the model with the lowest mean absolute error and the largest correlation coefficient values was considered the best model. The efficiency of developed ANN models for predicting the dependent variables (the quality of the prediction) was assessed through the *ex-post* measures of the prediction error, including the mean absolute error (MAE), the root mean square error (RMS), the relative approximation error (RAE), and the mean absolute percentage error (MAPE) statistics (Niedbała, 2019). The impact of independent variables (network inputs) on dependent variables was determined through a sensitivity analysis of the neural network. Error quotient and the rank of variables were applied in the sensitivity analysis (Niedbała et al., 2019a,b). A classical stepwise multiple regression model was generated with SAS® software using the same inputs and outputs of the ANN–MLP model.

## Results

The analysis of variance (ANOVA) reported a significant effect of the treatments and their interactions with the percentage of putative and PCR-verified transgenic plants (Table 1). The four-way interaction of *Agrobacterium* strain, *Agrobacterium* cell density, acetosyringone concentration, and inoculation duration was significant for the percentage of putative and PCR-verified transgenic tobacco plants at the 1% probability level (Table 1).

**Table 1:** Analysis of variance (ANOVA) of the percentage of putative and PCR-verified transgenic tobacco plants under the main and interactive effects of parameters investigated.

**Source of variation**	**degree of freedom**	Mean squares^*^	
		**Putative transgenes**	**PCR-verified transgenic plants**
*Agrobacterium* strain	2	1.800	18,009.565
*Agrobacterium* optical density (OD_600_)	2	0.238	2,387.563
Acetosyringone concentration	2	0.285	2,854.690
Inoculation Duration	2	0.320	3,202.952
*Agrobacterium* strain × OD_600_	4	0.072	723.022
*Agrobacterium* strain × Acetosyringone concentration	4	0.038	383.349
*Agrobacterium* strain × Inoculation duration	4	0.009	98.092
OD_600_ × Acetosyringone concentration	4	0.204	2,042.062
OD_600_ × Inoculation duration	4	0.032	329.166
Acetosyringone concentration × Inoculation duration	4	0.012	123.589
*Agrobacterium* strain × OD_600_ × Acetosyringone concentration	8	0.021	217.415
*Agrobacterium* strain × OD_600_ × Inoculation duration	8	0.005	59.192
*Agrobacterium* strain × Acetosyringone concentration × Inoculation duration	8	0.010	100.975
OD_600_ × Acetosyringone concentration × Inoculation duration	8	0.026	269.226
*Agrobacterium* strain × OD_600_ × Acetosyringone concentration × Inoculation duration	16	0.006	60.839
Coefficient of Variation (%)		16.770	16.770

* *Significant at 1% probability level*.

The best networks were selected, among 10,000 networks, according to the ideal qualitative indicators. The ANNs selected for further analysis were characterized by a linear aggregation function in all of the layers. A linear activation function was applied to the input layer. The neurons in the hidden layers included a hyperbolic activation function. Moreover, a logistic activation function was adopted in the output layer. Basic information on the quality of the adapted neural networks for modeling *Agrobacterium*-mediated gene transformation of tobacco is presented in Table 2. The small values of errors (learning, validation, test, average, deviation, and mean absolute errors) and the high amount of correlation obtained indicate the strength of the expanded models (Table 2).

**Table 2:** Basic information of the quality and structure of the neural models produced for putative and PCR-verified transgenic tobacco plants.

**Neural network structure**	**Putative transgenic plants**	**PCR-verified transgenic plants**
	**MLP 4:6-13-6-1:1**	**MLP 4:6-5-6-1:1**
Learning error	0.034	0.126
Validation error	0.047	0.084
Test error	0.035	0.209
Mean	0.233	6.744
Standard deviation	0.177	9.954
Average error	0.000	−0.031
Deviation error	0.036	5.721
Mean Absolute error	0.026	3.042
Quotient deviations	0.207	0.574
Correlation	0.978	0.819

**Figure 3:**
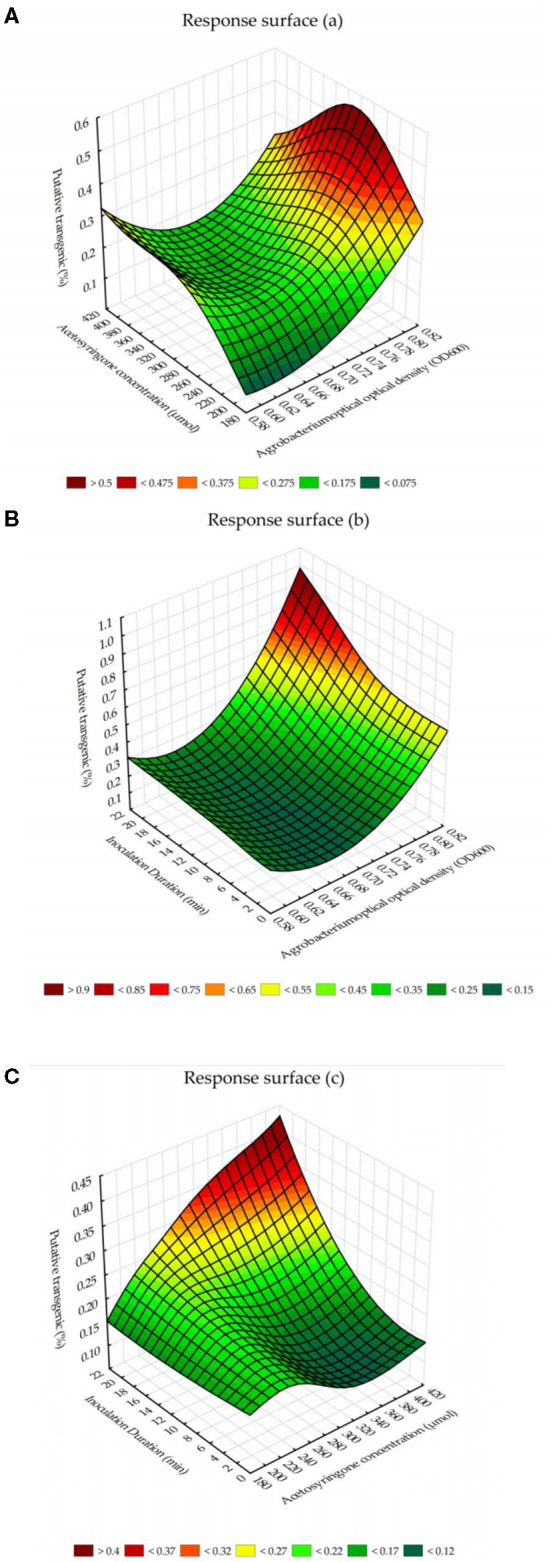
The response surface for the interaction effects of *Agrobacterium*-mediated gene transformation parameters on putative transgenic tobacco plants. **(A)** The interaction effects of *Agrobacterium* cell densities and acetosyringone concentrations on the percentage of putative transgenic tobacco plants. **(B)** The interaction effects of *Agrobacterium* cell densities and inoculation durations on the percentage of putative transgenic tobacco plants. **(C)** The interaction effects of acetosyringone concentrations and inoculation durations on the percentage of putative transgenic tobacco plants.

The response surface for the interaction effects of *Agrobacterium* cell densities and acetosyringone concentrations on putative transgenic tobacco plants showed that the highest percentage of putative transgenic tobacco plants can be obtained by the *Agrobacterium* cell density, at 600 nm, of 0.8 (*OD*_600_ = 0.8) × 280–300 μM of acetosyringone (Figure 3A). The chart for the response surface of the interaction effects of *Agrobacterium* cell densities and inoculation durations showed that *Agrobacterium* cell density of 0.8 (*OD*_600_ = 0.8) × 20 min inoculation is the best combination to reach the highest percentage of putative transgenic tobacco plants (Figure 3B). The chart for the response surface of the interaction effects of acetosyringone concentrations and inoculation durations showed that acetosyringone concentrations of 400 μM × 20 min inoculation is the best combination of these two variables in terms of percentage of the putative transgenic plants (Figure 3C).

**Figure 4:**
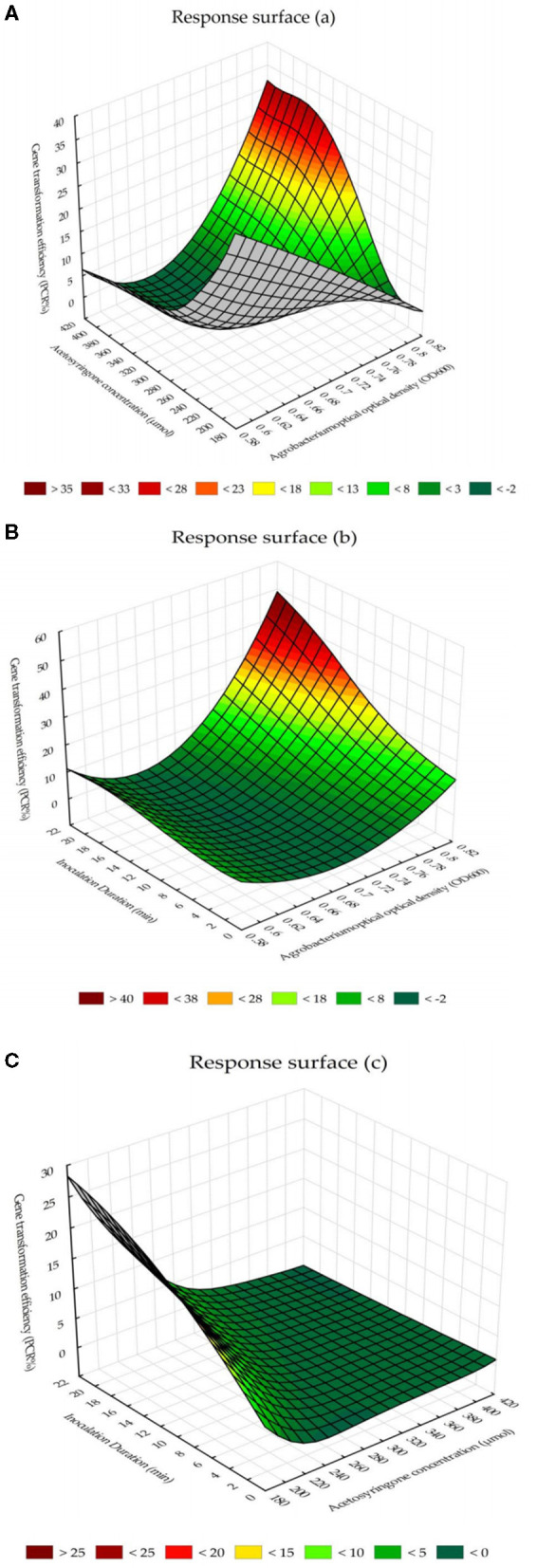
The response surface for the interaction effects of *Agrobacterium*-mediated gene transformation parameters on PCR-verified transgenic tobacco plants. **(A)** The interaction effects of *Agrobacterium* cell densities and acetosyringone concentrations on the percentage of transgenic tobacco plants. **(B)** The interaction effects of *Agrobacterium* cell densities and inoculation durations on the percentage of transgenic tobacco plants. **(C)** The interaction effects of acetosyringone concentrations and inoculation durations on the percentage of transgenic tobacco plants.

The response surface for the interaction effects of *Agrobacterium* cell densities and acetosyringone concentrations on PCR-verified plants showed the highest percentage of transgenic tobacco plants can be obtained by the *Agrobacterium* cell density, at 600 nm, of 0.8 (*OD*_600_ = 0.8) × 400 μM of acetosyringone (Figure 4A). The chart for the response surface of the interaction effects of *Agrobacterium* cell densities and inoculation durations showed that *Agrobacterium* cell density of 0.8 (*OD*_600_ = 0.8) × 20 min inoculation is the best combination to reach the highest percentage of transgenic tobacco plants (Figure 4B). The chart for the response surface of the interaction effects of acetosyringone concentrations and inoculation durations reported that acetosyringone concentrations of 220–240 μM × 18–20 min inoculation is the best combination of these two variables in terms of percentage of the PCR-verified transgenic plants (Figure 4C).

**Figure 5:**
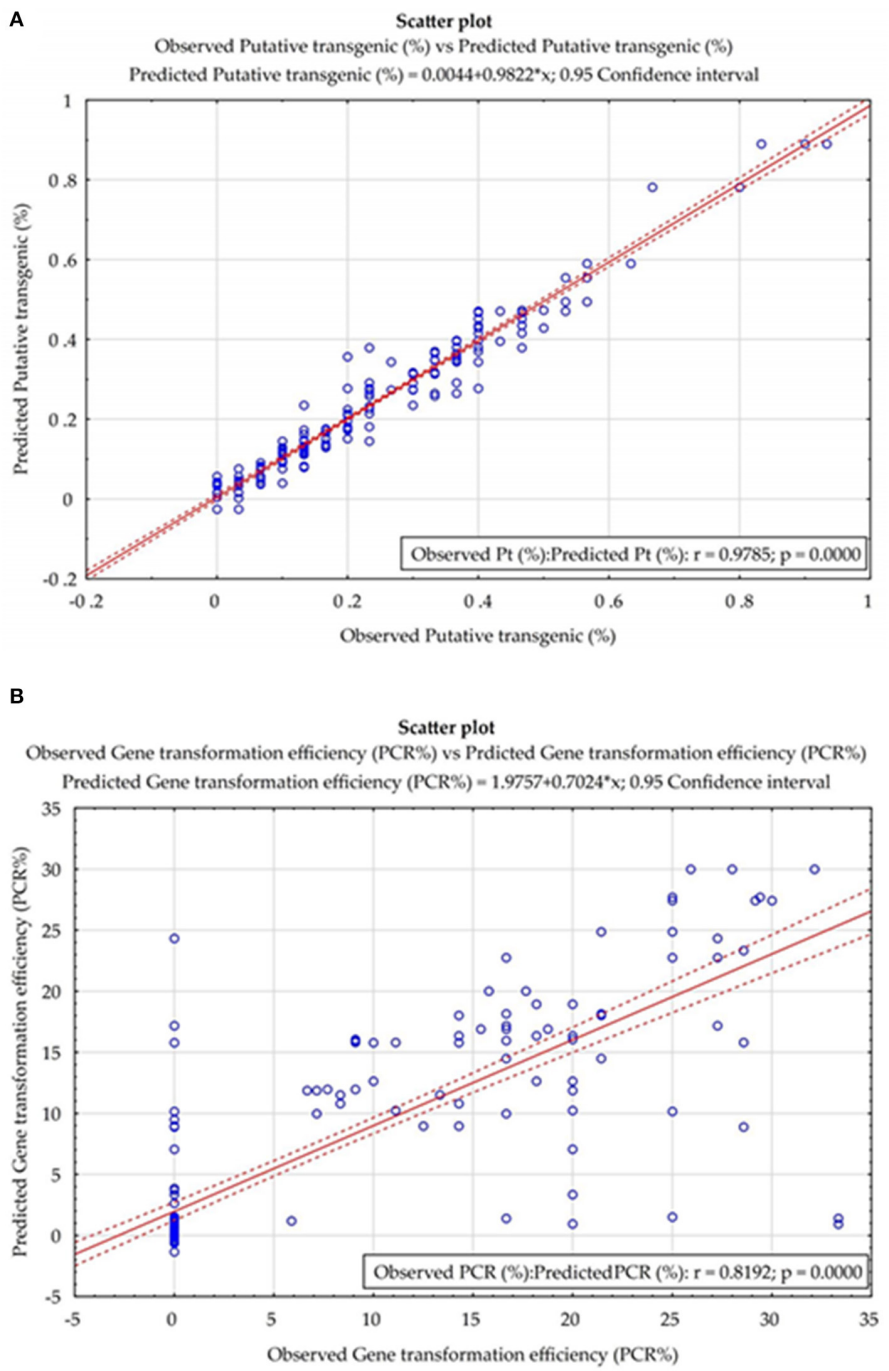
The scatter plot of the observed vs. the predicted values of **(A)** the percentage of putative transgenic tobacco plants and **(B)** the percentage of PCR-verified transgenic tobacco plants in the fitted ANN models.

The results of the prediction errors of the developed models, using measures prediction *ex-post* of analyzed neural models, are presented in Table 3. The small number of prediction errors (MAPE, RAE, RMS, and MAE) obtained, indicted the strength and accuracy of the developed models in forecasting the percentage of putative and PCR-verified transgenic tobacco plants (Table 3). The scatter plot of the observed vs. the predicted values of the percentage of putative transgenic tobacco plants showed there was no significant difference between the observed and the ANN–MLP predicted data (Figure 5A). The high value of the determination coefficient of the model (*R* = 0.97) suggests high repeatability of the established model (Figure 5A). The scatter plot of the observed vs. the predicted values of the percentage of PCR-verified transgenic tobacco plants reported no significant difference between the observed and the ANN–MLP predicted data (Figure 5B). The determination coefficient of the model (*R*^2^ = 0.97) indicates a good performance of the established model (*R* = 0.819) to predict the percentage of transgenic tobacco plants (Figure 5B).

**Table 3:** Measures prediction *ex-post* of analyzed neural models.

**Error measure**	**MAPE**	**RAE**	**RMS**	**MAE**
MLP 4:6-13-6-1:1	15.994	0.159	0.037	0.027
MLP 4:6-5-6-1:1	33.979	0.339	8.844	5.858

*MAPE, mean absolute percentage error; RAE, relative approximation error; RMS, root mean square error; MAE, mean absolute error*.

Comparison of the actual values of outputs, achieved from the interaction of the different levels of the four inputs, with predicted values achieved from the ANN models, showed the greatest measured and predicted values of the percentage of putative and PCR-verified transgenic tobacco plants were related to the interaction of the LB4404 strain of *Agrobacterium* × cell density of 0.8 (*OD*_600_ = 0.8) × 300 μM of acetosyringone × 20 min inoculation duration (Supplementary Tables 1, 2). Therefore, this combination of inputs was selected as the optimal condition for *Agrobacterium*-mediated transformation of tobacco. The next important combination of inputs that led to the higher measured and predicted values of the percentage of putative and PCR-verified transgenic tobacco plants was related to the GV3101 strain of *Agrobacterium* × cell density of 0.8 (*OD*_600_ = 0.8) × 300 μM of acetosyringone × 20 min inoculation duration (Supplementary Tables 1, 2). The lowest measured and predicted values of the putative transgenic plants were obtained from the AGL1 strain of *Agrobacterium* × cell density of 0.6 (*OD*_600_ = 0.6) × 400 μM of acetosyringone × 1 min inoculation duration and AGL1 strain of *Agrobacterium* × cell density of 0.7 (*OD*_600_ = 0.7) × 200 μM of acetosyringone × 1 min inoculation duration (Supplementary Table 1).

Results from the stepwise multiple regression analysis revealed the lower performance of this classical statistical model when compared to the ANN–MLP models, in terms of R^2^, RAE, MAE, RMS, and MAPE statistical values (Table 4). The correlation coefficient of the classical stepwise regression analysis for putative (*R*^2^ = 0.313) and PCR-verified transgenic plants (*R*^2^ = 0.213) was significantly lower than those obtained from MLP–ANN models (*R*^2^ = 0.956, *R*^2^ = 0.671). Instead, the prediction errors (MAPE, RAE, RMS, and MAE) of the stepwise multiple regression analysis for putative and PCR-verified transgenic plants (Table 4) were higher than those of the developed MLP–ANN models (Table 3).

**Table 4:** The performance of stepwise multiple regression to predict the percentage of putative and PCR-verified transgenic tobacco plants based on R^2^, MAPE, RAE, RMS, and MAE.

**Variable in the model**	**Output**	**Performance**
		R^2^	MAPE	RAE	RMS	MAE
*Agrobacterium* strain + *Agrobacterium* optical density + Acetosyringone concentration + Inoculation duration	Percentage of putative transgenic plants	0.313	18.834	0.267	0.149	0.124
	Percentage of PCR-verified transgenic plants	0.213	79.617	0.5000	8.922	19.990

The network sensitivity analysis of the produced neural models reported the highest rank (1) of investigated factors was related to the *Agrobacterium* strain, which suggests that this factor is the most important input variable with the potential to affect the percentage of putative transgenic tobacco plants (Table 5). Based on their impact on the *Agrobacterium*-mediated gene transformation of tobacco, *Agrobacterium* cell density, acetosyringone concentration, and inoculation duration were in the second, third, and fourth places of importance, respectively (Table 5).

**Table 5:** The sensitivity analysis of the governing variables on the percentage of putative and PCR-verified transgenic tobacco plants.

**Variable**	**Quotient**		**Rank**
	MLP 4:6-13-6-1:1	MLP 4:6-5-6-1:1	
*Agrobacterium* strain	4.172	2.109	1
*Agrobacterium* cell density (OD_600_)	3.596	1.718	2
Acetosyringone concentration	3.386	1.556	3
Inoculation duration	2.504	1.270	4

## Discussion

Earlier *Agrobacterium*-mediated transformation experiments were analyzed by classical statistical approaches, such as ANOVA and mean comparison analysis, to interpret the experimental results and establish an optimized *Agrobacterium*-mediated gene transformation protocol (Niazian et al., 2019; Gurusaravanan et al., 2020; Liu et al., 2020; Ma et al., 2020). In plant sciences, there are certain assumptions in order to utilize parametric analysis methods (e.g., ANOVA, correlation, *t*-tests, and regression). Normal distribution of residuals and homogeneity of variance of the errors are the most important assumptions (Ghasemi and Zahediasl, 2012). Checking data distribution, using the normality test (especially frequentist tests), is a prerequisite to apply parametric tests in plant data. However, in plant dynamic study, data have leptokurtic distribution and univariate normality tests are not efficient (Delmail et al., 2011). Therefore, non-parametric tests need to be applied when the basic mentioned assumptions are violated and there is evidence of non-normality and presence of outliers. Consequently, assumptions made on the distribution and homogeneity of the variance of errors are not required in non-parametric algorithms (Pour-Aboughadareh et al., 2019; Hocaoglu et al., 2020).

Results of this study reported that the highest levels of *Agrobacterium* optical density and acetosyringone concentration as well as inoculation duration led to an optimal response in the plant transformation. However, when all the experimental inputs are considered (four-way interactions), it was observed the highest level of acetosyringone did not lead to the highest predicted value of the outputs and the negative effect of the higher levels of acetosyringone was evident in the measured and the predicted values of putative and PCR-verified transgenic plants. Actually, we selected the effective levels of parameters investigated as the final level, and increased levels of these three inputs may lead to a lower percentage of transgenic plants. In biological experiments, such as *Agrobacterium*-mediated gene transformation, there is a threshold for the applied parameters. Levels and concentrations greater than the optimum will lead to an inhibitory effect. For example, higher *Agrobacterium* cell density and longer inoculation durations have destructive effects on host cells and significantly reduce regeneration. It is a similar situation for acetosyringone where concentrations higher than the optimum level have inhibitory effects on both *Agrobacterium* as well as the host cells (Niazian et al., 2019).

The efficiency of *Agrobacterium*-mediated gene transformation depends on the main and interactive effects of several pivotal parameters. Testing the serial levels (concentrations) of each parameter independently, and/or in combination with the serial levels of other parameters, is the methodology that has been applied to find the optimum level(s) of input and increase the efficiency of the *Agrobacterium*-mediated gene transformation strategies. However, this is a costly and time-consuming process. Predicting the value of the response from the values of the independent variables can help to understand the effect of a factor on a process (Barone, 2019). Usually, there are complex non-linear relationships that exist between the independent (input) and dependent (output) parameters of plant *in vitro* studies. In addition, the interaction of these parameters with the plant genotype and environment leads to a non-deterministic condition (Prasad and Gupta, 2008). Classical linear regression methods are unable to predict and interpret the non-linear and complex relationships between the variables investigated (Niazian et al., 2018). Non-parametric algorithms, such as ANNs, partial least square regression (PLSR), random forest (RF), and support vector machines (SVMs), have great efficiency for processing non-linear data (Zheng et al., 2018; Hesami and Jones, 2020; Niazian and Niedbała, 2020). Artificial neural networks, especially MLP, have superiority over the classical statistical methods for analyzing and interpreting unpredictable data sets (Salehi et al., 2020). As a data-driven model, ANN can be used for predicting and optimizing non-linear plant *in vitro* studies (Hesami et al., 2017). Through the full use of all spectral data and by avoiding multicollinearity, ANNs are able to manage non-normal, non-linear, and non-deterministic data sets obtained from multi-factorial plant *in vitro* studies. In MLP–ANN, the interconnection of neurons is formed by feedback on training with the backpropagation algorithm. Networks are trained to transform input data to a specific response (dependent variable). Interconnected neurons of hidden layers are not directly influenced by any input variable, which gives specific predictive power to the MLP–ANN (Barone, 2019). The scatter plot of the observed vs. the predicted values along with R^2^, RAE, MAE, RMS, and MAPE statistics demonstrated the predictive power and accuracy of the developed MLP–ANN models in this study. This accuracy was reflected in the close actual and predicted values of the percentage of putative and PCR-verified transgenic plants. The power of our developed models is reported as the prediction percentage of putative and PCR-verified transgenic tobacco plants. Tobacco is a model plant for gene transformation studies. The efficiency of the developed predictor model was tested in a medicinal plant with low transformation efficiency (Niazian et al., 2019). The results obtained showed a high similarity of the ANN-predicted and the actual values of the transformation efficiency in ajowan (*Trachyspermum ammi* L.), a medicinal plant (Supplementary Table 3). All similar actual and predicted values of the percentage of putative transgenic ajowan plants showed the efficiency of our predictor model in other plant species with low transformation efficiency. By using different data sets of the percentage of putative and/or PCR-verified transformants, other researchers can use these models in *Agrobacterium*-mediated transformation studies and predict the gene transformation efficiency of their protocols in desired dicot and monocot species.

The efficiency of an *Agrobacterium*-mediated gene transformation protocol can be determined through the percentage of regenerated plants in the selective medium, the molecular methods employed (percentage of PCR-verified plants and percentage of plants verified through advanced molecular techniques such as RT-PCR and Southern blot), and the expression methods used. In this study, we presented two different predictor models. The first one is a cost-effective predicting model, in *Agrobacterium*-mediated gene transformation of tobacco, as it used regenerated plants in the selective medium (putative transgenic plants) as output of the model and was found to be independent of time-consuming and expensive advanced molecular techniques. The regeneration percentage of inoculated explants in the selective medium is the most important factor that determines the efficiency of the final gene transformation study. The more the number of putative transgenic plants, the more the transgenic events. Finding and predicting the best combination(s) of factors influencing the percentage of putative transgenic lines will help increase the number of transgenic events in an *Agrobacterium*-mediated transformation study. Hence, the first model of this study is a fast and cost-effective model used to predict putative transformants in an *Agrobacterium*-mediated transformation study. However, the second model is suitable for predicting the transformation efficiency, as the output of this model is the percentage of PCR-verified plants. There is only one recently published paper in this field, which utilized gene transformation efficiency as output of the machine learning models. Actually, it is a data mining work that used an “ensemble model” for combining and mixing previously published data sets of *Agrobacterium*-mediated gene transformation of chrysanthemums employing different *in vitro* regeneration and *Agrobacterium*-mediated gene transformation protocols (Hesami et al., 2020a). The obtained high values of R^2^ and low values of prediction errors in developed ANN–MLP models over the classical stepwise multiple regression indicate the high similarities and low differences between the experimental data (observed) and the predicted values through the established ANN models and the superiority of the models in comparison with the classical model. The high similarity of the observed (measured) and the ANN-predicted data has been reported in earlier plant *in vitro* studies (Dutta Gupta and Pattanayak, 2017). With all the mentioned results, it should be noted that uncertainty is of major importance in machine learning algorithms for the purpose of prediction. Uncertainty caused by randomness (*aleatoric*) and uncertainty caused by ignorance (*epistemic*) are the two inherently different sources of uncertainty, which are usually not distinguishable in a learning algorithm. Additional data (information) can reduce the epistemic uncertainty in supervised learning algorithms (Hüllermeier and Waegeman, 2021).

The sensitivity analysis of the established ANN models showed the *Agrobacterium* strain is more important when compared to other parameters investigated in the gene transformation of tobacco. According to the sensitivity analysis on the developed ensemble model in *Agrobacterium*-mediated gene transformation of chrysanthemums, (Hesami and Jones, 2020) also reported the highest variable sensitivity ratio (1.86) for the *Agrobacterium* strain, Gehl et al. (2020) reported a better regeneration rate and potentially higher number of shoots in the genetic transformation of *Campanula medium* by the AGL1 *Agrobacterium* strain compared to the GV3101:pMP90 and ABI strains. The LB4404 *Agrobacterium* strain was more efficient than the AGL1 strain in the gene transformation of foxtail millet (*Setaria italic* L.) (Sood et al., 2020). Host–pathogen interaction is an important factor in *Agrobacterium*-mediated T-DNA delivery, as it can significantly affect the survival rate and regeneration activity of the transformed explants (Agarie et al., 2020). The transcription levels of the virulence (*vir*) genes in the induction medium can affect the transformation efficiency of *Agrobacterium tumefaciens* strains, as high transcriptional levels of *vir* genes were important for successful transformation (Wang et al., 2020).

It is obvious that there are other parameters that affect the efficiency of a gene delivery study. Parameters related to plasmids for optimizing expression (sub-optimal promoter, enhancer, poor codon usage, 5'UTR sequence, trigger silencing, integration of the gene into a silent region of chromatin) along with the *in vitro* regeneration parameters (type of the basal culture medium, explant type, explant age, type and concentration of PGRs, additives, etc.) are also involved in the results of an *Agrobacterium*-mediated transformation. Therefore, two sets of optimizations are needed to develop an efficient gene transformation protocol, including optimization of the tissue culture protocol parameters and optimization of the gene transformation protocol parameters, considering these parameters together can help to achieve a comprehensive and reliable model. However, this requires a huge number of experiments. In addition, there are some *in planta* transformation methods that are independent of the *in vitro* regeneration parameters (Niazian et al., 2017). Therefore, the results of this study are valuable for standard and *in planta Agrobacterium*-mediated transformation of plants.

## Conclusions

The optimization of plant *in vitro* studies, by taking into account all the influential factors, is laborious, time-consuming, and challenging because of its multi-factorial nature. A powerful data analysis can help researchers improve the efficiency, time, and cost-effectiveness of their techniques, and subsequently generate a better decision-making tool in complex biological processes. Using complex mathematical functions, ANNs are able to analyze non-deterministic and non-linear data sets of plant *in vitro* studies. Modeling and predicting complicated *in vitro* processes, such as *Agrobacterium*-mediated gene transformation, through ANNs can be useful in identifying the influencing factors. Establishing an optimized model in a specific plant genotype can be helpful in overcoming the barriers of genetic engineering in important *Agrobacterium*-recalcitrant plant genotypes.

This study demonstrates that a novel ANN is an accurate approach for assessing the effect of *Agrobacterium* strains, *Agrobacterium* cell densities, acetosyringone concentrations, and inoculation durations on the percentage of putative and PCR-verified transgenes in tobacco. Based on our results, the greatest actual and predicted values of the percentage of putative and PCR-verified transgenic plants were obtained by 20-min inoculation of tobacco leaf explants in a suspension of the LB4404 strain of *Agrobacterium* at optical density (OD_600_) of 0.8 and 300 μM of acetosyringone. The *Agrobacterium* strain was the most important factor among all parameters studied. The predicted values of the percentage of putative and PCR-verified transgenic plants were close to those observed, which indicates the efficiency of the established ANN models. The developed model was also efficient in predicting the gene transformation efficiency of an important medicinal plant with a low rate of transformation. Through the precise and efficient data interpretation, ANN could be helpful for optimizing the gene transformation conditions in *Agrobacterium*-mediated gene transformation studies, regarding all the influential parameters (expression plasmid optimization, *Agrobacterium*, and *in vitro* regeneration parameters), and open the way for targeted genome editing methods, such as clustered regularly interspaced short palindromic repeats-associated (CRISPR/Cas).

## Data Availability Statement

The original contributions presented in the study are included in the article/Supplementary Material, further inquiries can be directed to the corresponding author/s.

## Author Contributions

GN and MN conceptualized this study, curated the data, formal analysis, and validated this study. Investigation was carried out by MN. The methodology used in this study was framed by GN and MN, while MN handled the project administration and provided the resources. GN provided the software and visualization of the results. GN and MN wrote the original draft, reviewed, and edited it, while PS revised the manuscript for important intellectual content. All authors contributed to the article and approved the submitted version.

## Conflict of Interest

The authors declare that the research was conducted in the absence of any commercial or financial relationships that could be construed as a potential conflict of interest.

## Publisher's Note

All claims expressed in this article are solely those of the authors and do not necessarily represent those of their affiliated organizations, or those of the publisher, the editors and the reviewers. Any product that may be evaluated in this article, or claim that may be made by its manufacturer, is not guaranteed or endorsed by the publisher.

## Supplementary Material

Supplementary Table 1 The measured and the predicted values of the percentage of putative transgenic tobacco plants under the interaction effects of different levels of input.

Supplementary Table 2 The measured and the predicted values of the percentage of PCR-verified transgenic tobacco plants under the interaction effects of different levels of inputs.

Supplementary Table 3 The measured and the predicted values of the percentage of putative transgenic ajowan plants under the interaction effects of different levels of inputs.

## References

[B1] Abbasi H. Naderi R. Kafi M. Azadi P. Shakh-Asadi M. Okazaki K. (2020). Effect of ‘Chloroxynil' on *Agrobacterium*-mediated transformation efficiency of Lilium cv ‘Manissa.' Sci. Hortic. (Amsterdam) 271:109404. 10.1016/j.scienta.2020.109404

[B2] Agarie S. Umemoto M. Sunagawa H. Anai T. Cushman J. C. (2020). An *Agrobacterium*-mediated transformation via organogenesis regeneration of a facultative CAM plant, the common ice plant *Mesembryanthemum crystallinum* L. Plant Prod. Sci. 23, 343–349. 10.1080/1343943X.2020.1730700

[B3] Arab M. M. Yadollahi A. Eftekhari M. Ahmadi H. Akbari M. Khorami S. S. (2018). Modeling and optimizing a new culture medium for *in vitro* rooting of G × N15 prunus rootstock using artificial neural network-genetic algorithm. Sci. Rep. 8:9977. 10.1038/s41598-018-27858-4 29967468 PMC6028477

[B4] Barone J. O. (2019). Use of multiple regression analysis and artificial neural networks to model the effect of nitrogen in the organogenesis of *Pinus taeda* L. Plant Cell, Tissue Organ Cult. 137, 455–464. 10.1007/s11240-019-01581-y

[B5] Delmail D. Labrousse P. Botineau M. (2011). The most powerful multivariate normality test for plant genomics and dynamics data sets. Ecol. Inform. 6, 125–126. 10.1016/j.ecoinf.2011.01.003

[B6] Dutta Gupta S. Pattanayak A. K. (2017). Intelligent image analysis (IIA) using artificial neural network (ANN) for non-invasive estimation of chlorophyll content in micropropagated plants of potato. Vitr. Cell. Dev. Biol. - Plant 53, 520–526. 10.1007/s11627-017-9825-6

[B7] Emamgholizadeh S. Parsaeian M. Baradaran M. (2015). Seed yield prediction of sesame using artificial neural network. Eur. J. Agron. 68, 89–96. 10.1016/j.eja.2015.04.010

[B8] Gehl C. Li G. Serek M. (2020). An efficient protocol for *Agrobacterium*-mediated transformation and regeneration of Campanula medium (Canterbury bells) based on leaf disc explants. Plant Cell Tissue Organ Cult. 140, 635–645. 10.1007/s11240-019-01758-5

[B9] Ghasemi A. Zahediasl S. (2012). Normality tests for statistical analysis: a guide for non-statisticians. Int. J. Endocrinol. Metab. 10, 486–489. 10.5812/ijem.3505 23843808 PMC3693611

[B10] Gurusaravanan P. Vinoth S. Jayabalan N. (2020). An improved *Agrobacterium*-mediated transformation method for cotton (*Gossypium hirsutum* L. ‘KC3') assisted by microinjection and sonication. Vitr. Cell. Dev. Biol. Plant 56, 111–121. 10.1007/s11627-019-10030-6

[B11] Hesami M. Alizadeh M. Naderi R. Tohidfar M. (2020a). Forecasting and optimizing *Agrobacterium*-mediated genetic transformation via ensemble model- fruit fly optimization algorithm: a data mining approach using chrysanthemum databases. PLoS ONE 15:e0239901. 10.1371/journal.pone.0239901 32997694 PMC7526930

[B12] Hesami M. Jones A. M. P. (2020). Application of artificial intelligence models and optimization algorithms in plant cell and tissue culture. Appl. Microbiol. Biotechnol. 104, 9449–9485. 10.1007/s00253-020-10888-2 32984921

[B13] Hesami M. Naderi R. Tohidfar M. Yoosefzadeh-Najafabadi M. (2020b). Development of support vector machine-based model and comparative analysis with artificial neural network for modeling the plant tissue culture procedures: effect of plant growth regulators on somatic embryogenesis of chrysanthemum, as a case study. Plant Methods 16:112. 10.1186/s13007-020-00655-9 32817755 PMC7424974

[B14] Hesami M. Naderi R. Yoosefzadeh-Najafabadi M. Rahmati M. (2017). Data-driven modeling in plant tissue culture. J. Appl. Environ. Biol. Sci 7, 37–44.

[B15] Hocaoglu O. Akan K. Akçura M. (2020). Evaluating leaf rust reactions of pure bread wheat landrace lines using non-parametric statistics. Phytoparasitica 48, 261–271. 10.1007/s12600-019-00782-7

[B16] Hüllermeier E. Waegeman W. (2021). Aleatoric and epistemic uncertainty in machine learning: an introduction to concepts and methods. Mach. Learn. 110, 457–506. 10.1007/s10994-021-05946-3

[B17] Karmakar S. Molla K. A. Gayen D. Karmakar A. Das K. Sarkar S. N.. (2019). Development of a rapid and highly efficient *Agrobacterium*-mediated transformation system for pigeon pea [*Cajanus cajan* (L.) Millsp]. GM Crops Food 10, 115–138. 10.1080/21645698.2019.1625653 31187675 PMC6615537

[B18] Karthik S. Pavan G. Manickavasagam M. (2020). Nitric oxide donor regulates *Agrobacterium*-mediated genetic transformation efficiency in soybean [*Glycine max* (L.) Merrill]. Plant Cell Tissue Organ Cult. 141, 655–660. 10.1007/s11240-020-01808-3

[B19] Kujawa S. Nowakowski K. Tomczak R. J. Dach J. Boniecki P. Weres J.. (2014). Neural image analysis for maturity classification of sewage sludge composted with maize straw. Comput. Electron. Agric. 109, 302–310. 10.1016/j.compag.2014.08.014

[B20] Leng C. Sun B. Liu Z. Zhang L. Wei X. Zhou Y.. (2020). An optimized double T-DNA binary vector system for improved production of marker-free transgenic tobacco plants. Biotechnol. Lett. 42, 641–655. 10.1007/s10529-020-02797-1 31965394

[B21] Liu S. Ma J. Liu H. Guo Y. Li W. Niu S. (2020). An efficient system for *Agrobacterium*-mediated transient transformation in Pinus tabuliformis. Plant Methods 16:52. 10.1186/s13007-020-00594-5 32308730 PMC7149934

[B22] Ma R. Yu Z. Cai Q. Li H. Dong Y. Oksman-Caldentey K.-M.. (2020). *Agrobacterium*-mediated genetic transformation of the medicinal plant *Veratrum dahuricum*. Plants 9:191. 10.3390/plants9020191 32033134 PMC7076492

[B23] Meyers B. Zaltsman A. Lacroix B. Kozlovsky S. V. Krichevsky A. (2010). Nuclear and plastid genetic engineering of plants: comparison of opportunities and challenges. Biotechnol. Adv. 28, 747–756. 10.1016/j.biotechadv.2010.05.022 20685387

[B24] Murashige T. Skoog F. (1962). A revised medium for rapid growth and bio assays with tobacco tissue cultures. Physiol. Plant 15, 473–497. 10.1111/j.1399-3054.1962.tb08052.x

[B25] Mushtaq R. Shahzad K. Shah Z. H. Alsamadany H. Alzahrani H. A. S. Alzahrani Y.. (2020). Isolation of biotic stress resistance genes from cotton (*Gossypium arboreum*) and their analysis in model plant tobacco (*Nicotiana tabacum*) for resistance against cotton leaf curl disease complex. J. Virol. Methods 276:113760. 10.1016/j.jviromet.2019.113760 31712092

[B26] Niazian M. Niedbała G. (2020). Machine learning for plant breeding and biotechnology. Agriculture 10:436. 10.3390/agriculture10100436

[B27] Niazian M. Sadat Noori S. A. Galuszka P. Mortazavian S. M. M. (2017). Tissue culture-based *Agrobacterium*-mediated and in planta transformation methods. Czech J. Genet. Plant Breed. 53, 133–143. 10.17221/177/2016-CJGPB

[B28] Niazian M. Sadat-Noori S. A. Abdipour M. (2018). Modeling the seed yield of Ajowan (*Trachyspermum ammi* L.) using artificial neural network and multiple linear regression models. Ind. Crops Prod. 117, 224–234. 10.1016/j.indcrop.2018.03.013

[B29] Niazian M. Sadat-Noori S. A. Tohidfar M. Galuszka P. Mortazavian S. M. M. (2019). *Agrobacterium*-mediated genetic transformation of ajowan (*Trachyspermum ammi* (L.) Sprague): an important industrial medicinal plant. Ind. Crops Prod. 132, 29–40. 10.1016/j.indcrop.2019.02.005

[B30] Niedbała G. (2019). Simple model based on artificial neural network for early prediction and simulation winter rapeseed yield. J. Integr. Agric. 18, 54–61. 10.1016/S2095-3119(18)62110-0

[B31] Niedbała G. Nowakowski K. Rudowicz-Nawrocka J. Piekutowska M. Weres J. Tomczak R. J.. (2019a). Multicriteria prediction and simulation of winter wheat yield using extended qualitative and quantitative data based on artificial neural networks. Appl. Sci. 9:2773. 10.3390/app9142773

[B32] Niedbała G. Piekutowska M. Weres J. Korzeniewicz R. Witaszek K. Adamski M.. (2019b). Application of artificial neural networks for yield modeling of winter rapeseed based on combined quantitative and qualitative data. Agronomy 9:781. 10.3390/agronomy9120781

[B33] Pathi K. M. Tula S. Tuteja N. (2013). High frequency regeneration via direct somatic embryogenesis and efficient *Agrobacterium*—mediated genetic transformation of tobacco. Plant Signal. Behav. 8:e24354. 10.4161/psb.24354 23518589 PMC3906319

[B34] Pentoś K. (2016). The methods of extracting the contribution of variables in artificial neural network models—comparison of inherent instability. Comput. Electron. Agric. 127, 141–146. 10.1016/j.compag.2016.06.010

[B35] Pour-Aboughadareh A. Yousefian M. Moradkhani H. Poczai P. Siddique K. H. M. (2019). STABILITYSOFT: a new online program to calculate parametric and non-parametric stability statistics for crop traits. Appl. Plant Sci. 7:e01211. 10.1002/aps3.1211 30693157 PMC6342234

[B36] Prasad V. S. S. Gupta S. D. (2008). Applications and potentials of artificial neural networks in plant tissue culture;, in Plant Tissue Culture Engineering Focus on Biotechnology, Gupta S. D. Ibaraki Y.. (Dordrecht: Springer Netherlands).

[B37] Saini R. Sonia Jaiwal P. K. (2003). Stable genetic transformation of *Vigna mungo* L. Hepper via Agrobacterium tumefaciens. Plant Cell Rep. 22, 166–166. 10.1007/s00299-003-0643-4 12789502

[B38] Salehi M. Farhadi S. Moieni A. Safaie N. Ahmadi H. (2020). Mathematical modeling of growth and paclitaxel biosynthesis in *Corylus avellana* cell culture responding to fungal elicitors using multilayer perceptron-genetic algorithm. Front. Plant Sci. 11:1148. 10.3389/fpls.2020.01148 32849706 PMC7432144

[B39] Sika K. C. Kefela T. Adoukonou-Sagbadja H. Ahoton L. Saidou A. Baba-Moussa L.. (2015). A simple and efficient genomic DNA extraction protocol for large scale genetic analyses of plant biological systems. Plant Gene 1, 43–45. 10.1016/j.plgene.2015.03.001

[B40] Sood P. Singh R. K. Prasad M. (2020). An efficient *Agrobacterium*-mediated genetic transformation method for foxtail millet (*Setaria italica* L.). Plant Cell Rep. 39, 511–525. 10.1007/s00299-019-02507-w 31938834

[B41] Vasudevan V. Siva R. Krishnan V. Manickavasagam M. (2020). Polyamines, sonication and vacuum infiltration enhances the *Agrobacterium*-mediated transformation in watermelon (*Citrullus lanatus* Thunb.). S. Afr. J. Bot. 128, 333–338. 10.1016/j.sajb.2019.11.031

[B42] Wang S. Chen H. Wang Y. Pan C. Tang X. Zhang H.. (2020). Effects of *Agrobacterium tumefaciens* strain types on the *Agrobacterium*-mediated transformation efficiency of filamentous fungus *Mortierella alpina*. Lett. Appl. Microbiol. 70, 388–393. 10.1111/lam.13286 32077122

[B43] Wawrzyniak J. (2020). Application of artificial neural networks to assess the mycological state of bulk stored rapeseeds. Agriculture 10:567 10.3390/agriculture10110567

[B44] Zheng H. Li W. Jiang J. Liu Y. Cheng T. Tian Y.. (2018). A comparative assessment of different modeling algorithms for estimating leaf nitrogen content in winter wheat using multispectral images from an unmanned aerial vehicle. Remote Sens. 10:2026. 10.3390/rs10122026

