# Supplementary material for: Modeling Agrobacterium-Mediated Gene Transformation of Tobacco (Nicotiana tabacum)—A Model Plant for Gene Transformation Studies
Source: Front Plant Sci. 2021 Jul 23;12:695110. doi: 10.3389/fpls.2021.695110 (PMC8370025; doi:10.3389/fpls.2021.695110)
Supplement: Supplementary Table 1 — The measured and the predicted values of the percentage of putative transgenic tobacco plants under the interaction effects of different levels of input. [file Data_Sheet_1.docx]

Supplementary Material

**Table S1.** Measured and predicted values of percentage of putative transgenic plants of tobacco under interaction effect of different levels of inputs.

| Combination no. | Input | | | | Measured values | Predicted values |
| --- | --- | --- | --- | --- | --- | --- |
|  | Agrobacterium strain | Agrobacterium optical density (OD_600_) | Acetosyringone concentration (µmol) | Inoculation duration (min) |  |  |
| 1 | LB4404 | 0.6 | 200 | 1 | 0.155 | 0.133 |
| 2 | LB4404 | 0.6 | 200 | 10 | 0.100 | 0.118 |
| 3 | LB4404 | 0.6 | 200 | 20 | 0.166 | 0.138 |
| 4 | LB4404 | 0.6 | 300 | 1 | 0.144 | 0.173 |
| 5 | LB4404 | 0.6 | 300 | 10 | 0.177 | 0.175 |
| 6 | LB4404 | 0.6 | 300 | 20 | 0.200 | 0.180 |
| 7 | LB4404 | 0.6 | 400 | 1 | 0.111 | 0.118 |
| 8 | LB4404 | 0.6 | 400 | 10 | 0.133 | 0.162 |
| 9 | LB4404 | 0.6 | 400 | 20 | 0.166 | 0.165 |
| 10 | LB4404 | 0.7 | 200 | 1 | 0.133 | 0.132 |
| 11 | LB4404 | 0.7 | 200 | 10 | 0.200 | 0.195 |
| 12 | LB4404 | 0.7 | 200 | 20 | 0.222 | 0.214 |
| 13 | LB4404 | 0.7 | 300 | 1 | 0.111 | 0.123 |
| 14 | LB4404 | 0.7 | 300 | 10 | 0.133 | 0.133 |
| 15 | LB4404 | 0.7 | 300 | 20 | 0.200 | 0.224 |
| 16 | LB4404 | 0.7 | 400 | 1 | 0.133 | 0.126 |
| 17 | LB4404 | 0.7 | 400 | 10 | 0.177 | 0.151 |
| 18 | LB4404 | 0.7 | 400 | 20 | 0.233 | 0.225 |
| 19 | LB4404 | 0.8 | 200 | 1 | 0.177 | 0.172 |
| 20 | LB4404 | 0.8 | 200 | 10 | 0.233 | 0.232 |
| 21 | LB4404 | 0.8 | 200 | 20 | 0.422 | 0.435 |
| 22 | LB4404 | 0.8 | 300 | 1 | 0.344 | 0.347 |
| 23 | LB4404 | 0.8 | 300 | 10 | 0.466 | 0.467 |
| 24 | LB4404 | 0.8 | 300 | 20 | 0.888 | 0.890 |
| 25 | LB4404 | 0.8 | 400 | 1 | 0.388 | 0.397 |
| 26 | LB4404 | 0.8 | 400 | 10 | 0.322 | 0.313 |
| 27 | LB4404 | 0.8 | 400 | 20 | 0.355 | 0.370 |
| 28 | AGL1 | 0.6 | 200 | 1 | 0.055 | 0.039 |
| 29 | AGL1 | 0.6 | 200 | 10 | 0.022 | 0.037 |
| 30 | AGL1 | 0.6 | 200 | 20 | 0.100 | 0.097 |
| 31 | AGL1 | 0.6 | 300 | 1 | 0.066 | 0.090 |
| 32 | AGL1 | 0.6 | 300 | 10 | 0.133 | 0.148 |
| 33 | AGL1 | 0.6 | 300 | 20 | 0.211 | 0.180 |
| 34 | AGL1 | 0.6 | 400 | 1 | 0.000 | 0.014 |
| 35 | AGL1 | 0.6 | 400 | 10 | 0.066 | 0.053 |
| 36 | AGL1 | 0.6 | 400 | 20 | 0.122 | 0.129 |
| 37 | AGL1 | 0.7 | 200 | 1 | 0.000 | 0.004 |
| 38 | AGL1 | 0.7 | 200 | 10 | 0.011 | 0.002 |
| 39 | AGL1 | 0.7 | 200 | 20 | 0.033 | 0.056 |
| 40 | AGL1 | 0.7 | 300 | 1 | 0.022 | 0.032 |
| 41 | AGL1 | 0.7 | 300 | 10 | 0.077 | 0.039 |
| 42 | AGL1 | 0.7 | 300 | 20 | 0.066 | 0.075 |
| 43 | AGL1 | 0.7 | 400 | 1 | 0.011 | 0.016 |
| 44 | AGL1 | 0.7 | 400 | 10 | 0.033 | 0.002 |
| 45 | AGL1 | 0.7 | 400 | 20 | 0.022 | 0.039 |
| 46 | AGL1 | 0.8 | 200 | 1 | 0.100 | 0.094 |
| 47 | AGL1 | 0.8 | 200 | 10 | 0.055 | 0.047 |
| 48 | AGL1 | 0.8 | 200 | 20 | 0.111 | 0.079 |
| 49 | AGL1 | 0.8 | 300 | 1 | 0.111 | 0.112 |
| 50 | AGL1 | 0.8 | 300 | 10 | 0.122 | 0.116 |
| 51 | AGL1 | 0.8 | 300 | 20 | 0.388 | 0.380 |
| 52 | AGL1 | 0.8 | 400 | 1 | 0.088 | 0.082 |
| 53 | AGL1 | 0.8 | 400 | 10 | 0.011 | 0.042 |
| 54 | AGL1 | 0.8 | 400 | 20 | 0.111 | 0.111 |
| 55 | GV3101 | 0.6 | 200 | 1 | 0.200 | 0.209 |
| 56 | GV3101 | 0.6 | 200 | 10 | 0.155 | 0.145 |
| 57 | GV3101 | 0.6 | 200 | 20 | 0.277 | 0.277 |
| 58 | GV3101 | 0.6 | 300 | 1 | 0.311 | 0.264 |
| 59 | GV3101 | 0.6 | 300 | 10 | 0.266 | 0.257 |
| 60 | GV3101 | 0.6 | 300 | 20 | 0.422 | 0.415 |
| 61 | GV3101 | 0.6 | 400 | 1 | 0.311 | 0.317 |
| 62 | GV3101 | 0.6 | 400 | 10 | 0.266 | 0.273 |
| 63 | GV3101 | 0.6 | 400 | 20 | 0.344 | 0.365 |
| 64 | GV3101 | 0.7 | 200 | 1 | 0.344 | 0.349 |
| 65 | GV3101 | 0.7 | 200 | 10 | 0.422 | 0.451 |
| 66 | GV3101 | 0.7 | 200 | 20 | 0.488 | 0.473 |
| 67 | GV3101 | 0.7 | 300 | 1 | 0.300 | 0.291 |
| 68 | GV3101 | 0.7 | 300 | 10 | 0.344 | 0.343 |
| 69 | GV3101 | 0.7 | 300 | 20 | 0.455 | 0.470 |
| 70 | GV3101 | 0.7 | 400 | 1 | 0.244 | 0.235 |
| 71 | GV3101 | 0.7 | 400 | 10 | 0.277 | 0.275 |
| 72 | GV3101 | 0.7 | 400 | 20 | 0.544 | 0.494 |
| 73 | GV3101 | 0.8 | 200 | 1 | 0.388 | 0.395 |
| 74 | GV3101 | 0.8 | 200 | 10 | 0.433 | 0.428 |
| 75 | GV3101 | 0.8 | 200 | 20 | 0.555 | 0.554 |
| 76 | GV3101 | 0.8 | 300 | 1 | 0.444 | 0.467 |
| 77 | GV3101 | 0.8 | 300 | 10 | 0.588 | 0.590 |
| 78 | GV3101 | 0.8 | 300 | 20 | 0.711 | 0.781 |
| 79 | GV3101 | 0.8 | 400 | 1 | 0.388 | 0.379 |
| 80 | GV3101 | 0.8 | 400 | 10 | 0.311 | 0.356 |
| 81 | GV3101 | 0.8 | 400 | 20 | 0.422 | 0.434 |

**Table S2.** Measured and predicted values of percentage of PCR-verified transgenic plants of tobacco under interaction effect of different levels of inputs.

| Combination no. | Input | | | | Measured values | Predicted values |
| --- | --- | --- | --- | --- | --- | --- |
|  | Agrobacterium strain | Agrobacterium optical density (OD_600_) | Acetosyringone concentration (µmol) | Inoculation duration (min) |  |  |
| 1 | LB4404 | 0.6 | 200 | 1 | 0.000 | 0.000 |
| 2 | LB4404 | 0.6 | 200 | 10 | 0.000 | 0.000 |
| 3 | LB4404 | 0.6 | 200 | 20 | 1.111 | 0.920 |
| 4 | LB4404 | 0.6 | 300 | 1 | 0.000 | 0.000 |
| 5 | LB4404 | 0.6 | 300 | 10 | 2.222 | 2.632 |
| 6 | LB4404 | 0.6 | 300 | 20 | 3.333 | 3.353 |
| 7 | LB4404 | 0.6 | 400 | 1 | 0.000 | 0.000 |
| 8 | LB4404 | 0.6 | 400 | 10 | 0.000 | 0.000 |
| 9 | LB4404 | 0.6 | 400 | 20 | 0.000 | 0.000 |
| 10 | LB4404 | 0.7 | 200 | 1 | 0.000 | 0.000 |
| 11 | LB4404 | 0.7 | 200 | 10 | 0.000 | 0.000 |
| 12 | LB4404 | 0.7 | 200 | 20 | 0.000 | 0.000 |
| 13 | LB4404 | 0.7 | 300 | 1 | 0.000 | 0.000 |
| 14 | LB4404 | 0.7 | 300 | 10 | 0.000 | 0.000 |
| 15 | LB4404 | 0.7 | 300 | 20 | 0.000 | 0.000 |
| 16 | LB4404 | 0.7 | 400 | 1 | 0.000 | 0.000 |
| 17 | LB4404 | 0.7 | 400 | 10 | 0.000 | 0.000 |
| 18 | LB4404 | 0.7 | 400 | 20 | 0.000 | 0.000 |
| 19 | LB4404 | 0.8 | 200 | 1 | 0.000 | 0.000 |
| 20 | LB4404 | 0.8 | 200 | 10 | 0.000 | 0.000 |
| 21 | LB4404 | 0.8 | 200 | 20 | 7.777 | 7.805 |
| 22 | LB4404 | 0.8 | 300 | 1 | 5.555 | 5.780 |
| 23 | LB4404 | 0.8 | 300 | 10 | 8.888 | 9.925 |
| 24 | LB4404 | 0.8 | 300 | 20 | 25.555 | 27.777 |
| 25 | LB4404 | 0.8 | 400 | 1 | 5.555 | 6.666 |
| 26 | LB4404 | 0.8 | 400 | 10 | 4.444 | 5.555 |
| 27 | LB4404 | 0.8 | 400 | 20 | 0.000 | 0.000 |
| 28 | AGL1 | 0.6 | 200 | 1 | 0.000 | 0.000 |
| 29 | AGL1 | 0.6 | 200 | 10 | 0.000 | 0.000 |
| 30 | AGL1 | 0.6 | 200 | 20 | 0.000 | 0.000 |
| 31 | AGL1 | 0.6 | 300 | 1 | 0.000 | 0.000 |
| 32 | AGL1 | 0.6 | 300 | 10 | 0.000 | 0.000 |
| 33 | AGL1 | 0.6 | 300 | 20 | 0.000 | 0.000 |
| 34 | AGL1 | 0.6 | 400 | 1 | 0.000 | 0.000 |
| 35 | AGL1 | 0.6 | 400 | 10 | 0.000 | 0.000 |
| 36 | AGL1 | 0.6 | 400 | 20 | 1.111 | 1.518 |
| 37 | AGL1 | 0.7 | 200 | 1 | 0.000 | 0.000 |
| 38 | AGL1 | 0.7 | 200 | 10 | 0.000 | 0.000 |
| 39 | AGL1 | 0.7 | 200 | 20 | 0.000 | 0.000 |
| 40 | AGL1 | 0.7 | 300 | 1 | 0.000 | 0.000 |
| 41 | AGL1 | 0.7 | 300 | 10 | 0.000 | 0.000 |
| 42 | AGL1 | 0.7 | 300 | 20 | 0.000 | 0.000 |
| 43 | AGL1 | 0.7 | 400 | 1 | 0.000 | 0.000 |
| 44 | AGL1 | 0.7 | 400 | 10 | 0.000 | 0.000 |
| 45 | AGL1 | 0.7 | 400 | 20 | 0.000 | 0.000 |
| 46 | AGL1 | 0.8 | 200 | 1 | 0.000 | 0.000 |
| 47 | AGL1 | 0.8 | 200 | 10 | 0.000 | 0.000 |
| 48 | AGL1 | 0.8 | 200 | 20 | 1.111 | 1.518 |
| 49 | AGL1 | 0.8 | 300 | 1 | 0.000 | 0.000 |
| 50 | AGL1 | 0.8 | 300 | 10 | 0.000 | 0.000 |
| 51 | AGL1 | 0.8 | 300 | 20 | 8.888 | 9.982 |
| 52 | AGL1 | 0.8 | 400 | 1 | 0.000 | 0.000 |
| 53 | AGL1 | 0.8 | 400 | 10 | 0.000 | 0.000 |
| 54 | AGL1 | 0.8 | 400 | 20 | 0.000 | 0.000 |
| 55 | GV3101 | 0.6 | 200 | 1 | 0.000 | 0.000 |
| 56 | GV3101 | 0.6 | 200 | 10 | 2.222 | 2.798 |
| 57 | GV3101 | 0.6 | 200 | 20 | 3.333 | 3.854 |
| 58 | GV3101 | 0.6 | 300 | 1 | 5.555 | 6.753 |
| 59 | GV3101 | 0.6 | 300 | 10 | 2.222 | 2.798 |
| 60 | GV3101 | 0.6 | 300 | 20 | 7.777 | 7.074 |
| 61 | GV3101 | 0.6 | 400 | 1 | 4.444 | 7.074 |
| 62 | GV3101 | 0.6 | 400 | 10 | 2.222 | 3.854 |
| 63 | GV3101 | 0.6 | 400 | 20 | 6.666 | 3.854 |
| 64 | GV3101 | 0.7 | 200 | 1 | 5.555 | 7.074 |
| 65 | GV3101 | 0.7 | 200 | 10 | 4.444 | 4.782 |
| 66 | GV3101 | 0.7 | 200 | 20 | 5.555 | 7.074 |
| 67 | GV3101 | 0.7 | 300 | 1 | 4.444 | 5.732 |
| 68 | GV3101 | 0.7 | 300 | 10 | 5.555 | 5.999 |
| 69 | GV3101 | 0.7 | 300 | 20 | 7.777 | 9.494 |
| 70 | GV3101 | 0.7 | 400 | 1 | 0.000 | 1.453 |
| 71 | GV3101 | 0.7 | 400 | 10 | 0.000 | 1.453 |
| 72 | GV3101 | 0.7 | 400 | 20 | 1.111 | 1.453 |
| 73 | GV3101 | 0.8 | 200 | 1 | 3.333 | 4.353 |
| 74 | GV3101 | 0.8 | 200 | 10 | 4.444 | 4.353 |
| 75 | GV3101 | 0.8 | 200 | 20 | 15.555 | 15.962 |
| 76 | GV3101 | 0.8 | 300 | 1 | 4.444 | 4.353 |
| 77 | GV3101 | 0.8 | 300 | 10 | 10.000 | 11.957 |
| 78 | GV3101 | 0.8 | 300 | 20 | 20.000 | 18.938 |
| 79 | GV3101 | 0.8 | 400 | 1 | 11.111 | 12.635 |
| 80 | GV3101 | 0.8 | 400 | 10 | 6.666 | 8.956 |
| 81 | GV3101 | 0.8 | 400 | 20 | 10.000 | 11.957 |

**Table S3.** Measured and predicted values of percentage of putative transgenic plants of ajowan under interaction effect of different levels of inputs.

| Combination no. | Input | | | | Measured values | Predicted values |
| --- | --- | --- | --- | --- | --- | --- |
|  | Agrobacterium strain | Agrobacterium optical density (OD_600_) | Acetosyringone concentration (µmol) | Inoculation duration (min) |  |  |
| 1 | LB4404 | 0.2 | 200 | 1 | 0.000 | 0.012 |
| 2 | LB4404 | 0.2 | 200 | 20 | 0.000 | 0.012 |
| 3 | LB4404 | 0.2 | 200 | 30 | 0.333 | 0.115 |
| 4 | LB4404 | 0.2 | 250 | 1 | 0.000 | 0.135 |
| 5 | LB4404 | 0.2 | 250 | 20 | 0.000 | 0.135 |
| 6 | LB4404 | 0.2 | 250 | 30 | 3.333 | 3.666 |
| 7 | LB4404 | 0.2 | 300 | 1 | 0.333 | 0.319 |
| 8 | LB4404 | 0.2 | 300 | 20 | 0.000 | 0.174 |
| 9 | LB4404 | 0.2 | 300 | 30 | 0.000 | 0.174 |
| 10 | LB4404 | 0.2 | 400 | 1 | 0.000 | 0.174 |
| 11 | LB4404 | 0.2 | 400 | 20 | 0.000 | 0.174 |
| 12 | LB4404 | 0.2 | 400 | 30 | 0.000 | 0.174 |
| 13 | LB4404 | 0.8 | 200 | 1 | 3.333 | 3.385 |
| 14 | LB4404 | 0.8 | 200 | 20 | 7.666 | 8.069 |
| 15 | LB4404 | 0.8 | 200 | 30 | 8.000 | 8.069 |
| 16 | LB4404 | 0.8 | 250 | 1 | 9.333 | 9.745 |
| 17 | LB4404 | 0.8 | 250 | 20 | 11.000 | 13.324 |
| 18 | LB4404 | 0.8 | 250 | 30 | 21.666 | 25.666 |
| 19 | LB4404 | 0.8 | 300 | 1 | 13.666 | 14.047 |
| 20 | LB4404 | 0.8 | 300 | 20 | 12.666 | 12.789 |
| 21 | LB4404 | 0.8 | 300 | 30 | 17.666 | 18.987 |
| 22 | LB4404 | 0.8 | 400 | 1 | 5.666 | 6.785 |
| 23 | LB4404 | 0.8 | 400 | 20 | 5.000 | 5.777 |
| 24 | LB4404 | 0.8 | 400 | 30 | 5.333 | 5.734 |
| 25 | LB4404 | 1.0 | 200 | 1 | 3.333 | 3.745 |
| 26 | LB4404 | 1.0 | 200 | 20 | 4.333 | 4.798 |
| 27 | LB4404 | 1.0 | 200 | 30 | 3.666 | 3.714 |
| 28 | LB4404 | 1.0 | 250 | 1 | 4.333 | 7.417 |
| 29 | LB4404 | 1.0 | 250 | 20 | 4.666 | 0.806 |
| 30 | LB4404 | 1.0 | 250 | 30 | 11.333 | 11.777 |
| 31 | LB4404 | 1.0 | 300 | 1 | 0.000 | 0.317 |
| 32 | LB4404 | 1.0 | 300 | 20 | 0.000 | 0.317 |
| 33 | LB4404 | 1.0 | 300 | 30 | 2.666 | 0.806 |
| 34 | LB4404 | 1.0 | 400 | 1 | 0.000 | 0.317 |
| 35 | LB4404 | 1.0 | 400 | 20 | 0.000 | 0.317 |
| 36 | LB4404 | 1.0 | 400 | 30 | 0.000 | 0.317 |
| 37 | GV3101 | 0.2 | 200 | 1 | 0.000 | 0.317 |
| 38 | GV3101 | 0.2 | 200 | 20 | 0.000 | 0.317 |
| 39 | GV3101 | 0.2 | 200 | 30 | 0.000 | 0.317 |
| 40 | GV3101 | 0.2 | 250 | 1 | 0.000 | 0.317 |
| 41 | GV3101 | 0.2 | 250 | 20 | 0.000 | 0.317 |
| 42 | GV3101 | 0.2 | 250 | 30 | 0.000 | 0.317 |
| 43 | GV3101 | 0.2 | 300 | 1 | 0.000 | 0.317 |
| 44 | GV3101 | 0.2 | 300 | 20 | 0.000 | 0.317 |
| 45 | GV3101 | 0.2 | 300 | 30 | 0.000 | 0.317 |
| 46 | GV3101 | 0.2 | 400 | 1 | 0.000 | 0.317 |
| 47 | GV3101 | 0.2 | 400 | 20 | 0.000 | 0.317 |
| 48 | GV3101 | 0.2 | 400 | 30 | 0.000 | 0.317 |
| 49 | GV3101 | 0.8 | 200 | 1 | 1.000 | 0.317 |
| 50 | GV3101 | 0.8 | 200 | 20 | 2.000 | 1.333 |
| 51 | GV3101 | 0.8 | 200 | 30 | 1.666 | 1.777 |
| 52 | GV3101 | 0.8 | 250 | 1 | 2.000 | 2.317 |
| 53 | GV3101 | 0.8 | 250 | 20 | 3.000 | 3.656 |
| 54 | GV3101 | 0.8 | 250 | 30 | 6.666 | 7.012 |
| 55 | GV3101 | 0.8 | 300 | 1 | 2.000 | 2.312 |
| 56 | GV3101 | 0.8 | 300 | 20 | 3.000 | 3.098 |
| 57 | GV3101 | 0.8 | 300 | 30 | 4.666 | 4.788 |
| 58 | GV3101 | 0.8 | 400 | 1 | 0.000 | 0.012 |
| 59 | GV3101 | 0.8 | 400 | 20 | 0.000 | 0.012 |
| 60 | GV3101 | 0.8 | 400 | 30 | 2.333 | 2.555 |
| 61 | GV3101 | 1.0 | 200 | 1 | 0.666 | 0.971 |
| 62 | GV3101 | 1.0 | 200 | 20 | 1.000 | 1.111 |
| 63 | GV3101 | 1.0 | 200 | 30 | 1.333 | 1.667 |
| 64 | GV3101 | 1.0 | 250 | 1 | 1.333 | 1.667 |
| 65 | GV3101 | 1.0 | 250 | 20 | 2.000 | 2.555 |
| 66 | GV3101 | 1.0 | 250 | 30 | 4.333 | 4.445 |
| 67 | GV3101 | 1.0 | 300 | 1 | 3.000 | 3.333 |
| 68 | GV3101 | 1.0 | 300 | 20 | 0.000 | 0.012 |
| 69 | GV3101 | 1.0 | 300 | 30 | 0.000 | 0.012 |
| 70 | GV3101 | 1.0 | 400 | 1 | 0.000 | 0.012 |
| 71 | GV3101 | 1.0 | 400 | 20 | 0.000 | 0.012 |
| 72 | GV3101 | 1.0 | 400 | 30 | 0.000 | 0.012 |
